# A Review of Actuation and Sensing Mechanisms in MEMS-Based Sensor Devices

**DOI:** 10.1186/s11671-021-03481-7

**Published:** 2021-01-26

**Authors:** Abdullah Saleh Algamili, Mohd Haris Md. Khir, John Ojur Dennis, Abdelaziz Yousif Ahmed, Sami Sultan Alabsi, Saeed Salem Ba Hashwan, Mohammed M. Junaid

**Affiliations:** 1 Department of Electrical and Electronic Engineering, Universiti Teknologi PETRONAS, 32610 Seri Iskandar, Malaysia; 2 Department of Fundamental and Applied Sciences, Universiti Teknologi PETRONAS, 32610 Seri Iskandar, Malaysia

**Keywords:** Actuators, Capacitive, Electromagnetic, Electrostatic, Electrothermal, Fabrication, MEMS, Piezoelectric, Piezoresistive and sensors

## Abstract

Over the last couple of decades, the advancement in Microelectromechanical System (MEMS) devices is highly demanded for integrating the economically miniaturized sensors with fabricating technology. A sensor is a system that detects and responds to multiple physical inputs and converting them into analogue or digital forms. The sensor transforms these variations into a form which can be utilized as a marker to monitor the device variable. MEMS exhibits excellent feasibility in miniaturization sensors due to its small dimension, low power consumption, superior performance, and, batch-fabrication. This article presents the recent developments in standard actuation and sensing mechanisms that can serve MEMS-based devices, which is expected to revolutionize almost many product categories in the current era. The featured principles of actuating, sensing mechanisms and real-life applications have also been discussed. Proper understanding of the actuating and sensing mechanisms for the MEMS-based devices can play a vital role in effective selection for novel and complex application design.

## Introduction

Sensors and actuators are collectively stated as transducers, which serve the function of transforming signals or power from one energy domain to another [1, 2]. A wide range of transduction instruments are to convert physical signals into electrical signals (i.e., sensors). Besides, the output signals further processed by electronic systems using integrated circuits (ICs), just like converting electrical signals into physical signals (i.e., actuators) [3]. Particularly, sensors are the devices that detect and monitor physical phenomenon (i.e. vibration, pressure, and flow) or composition variations (electrical conductivity and potential hydrogen (pH)). Sensors convert variations into a particular form that can be utilized to mark or control measured variables [4, 5], whereas actuators are utilized to produce mechanical motion and force/torque. In another word, sensing can be broadly defined as energy transduction processes that result in perception, whereas actuation is energy transduction processes that produce actions. Sensors consist of three parts; sensing element to detect the physical and chemical quantity, transducer to convert the detected parameter to an electrical signal, readout device such as a computer that is used to read and interpret the converted signal.

The performance of sensors has been evaluated by various characteristic parameters, such as sensitivity, resolution, and accuracy etc. Whereas sensitivity determines the minimum value of the target substance concentration. Resolution refers to a ratio between the maximum magnitudes measured to the smallest part that can be determined. At the same time, accuracy is defined as the amount of uncertainty in measurement with respect to an absolute standard, and it can directly influence the qualitative analysis of the sensor [6]. Whereas the limit of detection (LOD) is the lowest quantity of a substance that can be distinguished by the sensor, wherein the capability of a sensor to identify a particular substance. In addition, the response time is the particular time period when the concentration reaches a certain limit when the sensor produces a warning signal and the recovery time is the period after the detection process for the sensing material that takes to recover and restore its baseline status.

In the past few decades, advances in microelectronic device fabrication technologies have produced compelling, accurate and high-performance device systems. Technology has been squeezed to the point where we can make devices so tiny that they are not noticeable to the human eye. Microelectromechanical systems (MEMS) involve the innovation of the tiny devices that can represent the models as sensors or actuators. Continuous development in the field of MEMS holds a promise for the optimized and cost saving miniaturized electronic equipment [7–9]. Typical dimensions of MEMS devices are generally measured in tens or hundreds of microns. Utilizing the same fabricating methods becomes like constructing microprocessors. Currently, sensors and actuators can be constructed at a similar scale level with the microprocessor chips. In recent developments, microscale batch fabrication of pressure, temperature, inertial etc., sensors have been demonstrated for batch scale using similar chip handling unit. The accurately superimposed a system on a chip can enable the operation of complex systems [10, 11].

MEMS is a technology that is fabricated using semiconductor materials and incorporates mechanical components, sensors, actuators, and electronic elements on a general silicon substrate with feature sizes grading from a few millimetres to microns gauge [12]. These systems can perform the operation of sensing, controlling and actuating at microscale, which can operate either individually or in bulk to produce an effect on the macro scale. MEMS technology has considered the combination of the microelectronics with micromachining technology on a typical Si wafer into typical metal oxide semiconducting devices [13]. In recent times, MEMS technology has been grown significantly for acknowledging different sorts of natural sensors and actuators. Besides, it has been utilized in miniaturized sensor manufacturing in a large number of applications due to low power ratings [14], quick response, array fabrication at mechanical measures, ease, cheap and get better sensitivity. Significantly, the surface to volume ration of sensing material have been increased substantially, which has ultimately reduced the operating temperature of the metal oxide-based sensor and have increased the demand for expansion materials [15–17].

Recently, metal oxide-based sensors such as titanium dioxide (TiO_2_), tin dioxide (SnO_2_) and zinc oxide (ZnO) has become the most attractive type of sensors for the detection of gases [18]. The working principle of the metal oxide semiconductor (MOS) technique depends on the change in resistivity of a metal oxide semiconductor used as an acceptor material when exposed to an analyte gas during detection [19]. However, the high sensitivity of these sensors would only be achieved at elevated operating temperatures [20], in addition there is also selectivity issues. An alternative approach is to combine it with other sensing components, particularly noble metal nanoparticles (i.e. Au, Ag, Pd, and Pt), to overcome the barriers mentioned above, due to the extreme specific advantages of chemical and electronic sensitization [21–23].

MEMS devices can be fabricated in the MEMSCAP US through the MUMPs (Multi-User MEMS) procedures. MUMPs is a profitmaking program that gives practical, evidence of-idea MEMS creation to industrial and academic research. Overall MEMSCAP provides three standard procedures as a significant aspect of the MUMPs package: PolyMUMPs, described as a three-layer polysilicon surface microfabrication procedure, MetalMUMPs, that is an electroplated nickel procedure, and SOIMUMPs, presented in a silicon-on-insulator microfabrication procedure [24]. MEMS procedures have developed from the unique technologies of semiconductor device fabrication: deposition, patterning, and etching of material layers [7, 25]. The steps of MUMPs fabrication technology are shown in the design handbook rule [24]. Despite fabrication technology, MEMS sensors have been broadly used as instrumentation or human comfort issues in industrial and home applications. Because of the diverse working condition of MEMS sensors in the different application fields (from internal use to outdoor utilizes), different sorts of MEMS sensors have been modelled based on various working principles and different sensor materials. The MEMS sensors are essential in gas sensing systems, which includes humidity and toxic gas sensors [19].

MEMS is a broad domain and almost covering each aspect of our present life, as sensors and actuators. The emerging domains of electronic microsystems, including almost all product categories in miniaturized forms, which has contributed a rapid development in the field of actuation and sensing techniques in their production and integration processes. The efficacy of a targeted application is critically dependent on the proper selection of a specific actuator. In addition, the basic efficiency of the actuator depends on different factors such as power and control methods, compatibility, the degree of packaging needed and cost-effectiveness. This article provides a thorough analysis of the MEMS actuation and sensing technologies investigated for their functional applications, with an emphasis on many common forms of transduction, in order to promote the advancement of this emerging area while addressing these main factors. The rest of the paper is arranged as follows. Section II introduces the Micro-Electro-Mechanical Systems. Section III presents a brief review of the actuation mechanisms (different principles and approaches to actuate MEMS devices include: electrostatic, electrothermal, electromagnetic, and piezoelectric actuation). Section IV introduces a brief overview of the sensing mechanisms include piezoresistive, capacitive and optical sensing mechanisms, and section V present a further discussion about the prospects of MEMS-based devices, finally, section VI provide the conclusions and future trends.

## Micro-electro-Mechanical Systems

The first appearance of what is known today as MEMS technology can be traced back to April 1, 1954, when Smith (1954) published a paper in the Bell Telephone Lab as a Physical Review. This is the first description of some of the stress-sensitive effects in silicon and germanium, called piezoresistors [26]. MEMS is not the main abbreviation that is especially reasonable because of the huge expansiveness and assortment of devices and systems that have being miniaturized (i.e., the field is not just micro, electrical and mechanical systems). Nevertheless, the abbreviation MEMS is most commonly utilized to indicate to the whole field (i.e., all devices resulting from micromachining other than IC), also written as micro-electro-mechanical, Microelectromechanical or microelectronic and MEMS in the United States) [3]. Different names for this general area of miniaturization involve Microsystems Technology (MST) famous in Europe [27], and Micromechanics famous in Asia [28]. MEMS devices involve a broad scope of domains to measure information from the surrounding environment and convert it to useful electrical signals. There are six major energy domains of interests:

1. Electrical domain (involve electric field, current, voltage, resistance, charge, capacitance, inductance, dielectric constant, polarization and frequency).
2. Chemical domain (include composition, reaction rate, concentration, pH and oxidation or reduction potential).
3. Mechanical domain (involve length, width, area, all-time derivatives such as velocity, acceleration, mass flow, volume, force, pressure, torque, acoustic wavelength and acoustic intensity).
4. Thermal domain (include temperature, flow, heat, specific heat, entropy and state of any matter).
5. Radiative domain (involve intensity, phase, polarization, wavelength, reflectance, transmittance and refractive index).
6. Magnetic domain (include field intensity, permeability, flux density and magnetic moment).

These energy domains and commonly encountered parameters within them are summarized in (Table 1). The total energy within a system can coexist in several domains and can shift among various domains under the right circumstances [1, 29].

**Table 1:** Energy domain analogies

Nature	Chemical domain	Electrical domain	Mechanical domain	Thermal domain	Radiative domain	Magnetic domain
Potential	Chemical concentration	Voltage	Force	Temperature	Electromagnetic waves	Magnetic field strength
Flow	Reaction rate	Current	Velocity	Entropy flow rate	Infrared radiation	Magnetic direction
Generalized displacement	Molecule recognition	Charge	Displacement	Entropy	Transmission	Electromagnetic force
Generalized resistance	DNA sequence	Resistance	Damping	Thermal resistance	UV radiation	Lorentz force
Generalized inductance	DNA hybridization	Inductance	Mass	–	X-rays	Induction
Generalized capacitance	Protein construct	Capacitance	Mechanical compliance	Thermal capacitance	Absorption	–

**Fig. 1:**
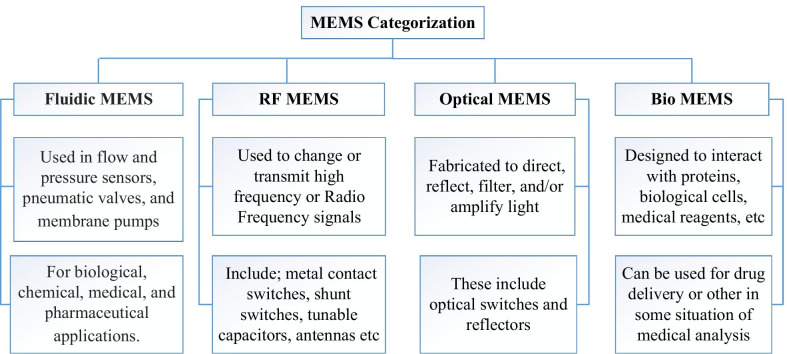
Categorization of MEMS devices

According to the operation of the designed chip, MEMS can be divided into different categories. It can be used to execute the sensing for measuring, monitoring and detecting hazardous gases. Another classification is using MEMS as action type device which act upon the body or with the body materials, like fluids or acting outside the body, such as devices used in drug delivery systems. MEMS devices have been incorporated into four distinct categories based on the core application areas (Fig. 1), which include: fluidic, Radio frequency (RF), optical, and bio MEMS [10, 30–33].

For most of the present technologies, MEMS sensors are a preferred new field for many practical applications starts from consumer electronics to the aeronautics industry. The most important feature of MEMS is the capability to communicate effectively with electrical components in semiconductor chips, and the sensor industry has been revolutionized using MEMS technology by combining electronic functions and mechanical actions [34], they normally have low power consumption and high sensitivity due to the small size [35, 36]. MEMS-based sensor devices have many advantages that make them play an important role in various applications. These advantages include low cost owing to the potentiality of array fabrication [37, 38], low power consumption [37–40] and small size [38–42]. Also, MEMS has many other advantages, such as lightweight, high resolution, stable performance and ease of integration with other devices and systems. Micromechanical to micromechanical device reductions have been improved in several areas, for instance, inertial sensors, chemical sensors, inkjet printers, gyroscopes, satellites, RF communications, smartphones, pressure sensors, accelerometers, biomedical instrumentation [43], military applications, motion and force sensors [44]. Furthermore, the low cost and simplicity of the fabrication process play a crucial role in commercial manufacturing [45].

According to the working principle, sensor devices can be divided into two categories based on the working principle: static and dynamic mode of the device operation [46, 47]. When the MEMS devices are based on the static mode, a frequency signal is not included. Also, the beam is deflected owing to the surface adsorption mass, which will cause temporary results of stress and structural deformation. It can be optically detected or sensed by changes in the piezoresistor. Certain chemical bonding arises on the device surface, and the internal Nano mechanics can bend the MEMS beam detection [46]. In the dynamic mode, to get optimum performance of the device, a mechanical resonant frequency should be stimulated. The maximum amplitude that can be achieved by the vibrating system and excite a maximum resonance in a specific system is called a resonance frequency. In which the resonant frequency is affected by two main parameters: the spring constant of the beam and its effective mass, and will change according to these parameters. This is the working principle for utilizing the change in resonance frequency as detection means. The quality factor has to be high to obtain the desired sensitivity and resolution [48, 49]. It is necessary to understand and compare these mechanisms since they are the basis of the MEMS-based devices operation and output signal detection**.**

## Actuation Techniques

**Fig. 2:**
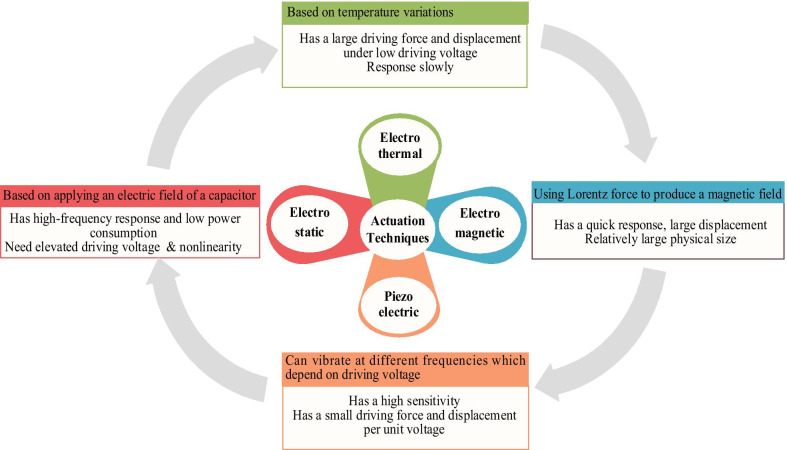
Actuation techniques of MEMS-based sensor device

The actuation technique is a term given to the mechanism that transforms the input energy into a microstructure motion. There are different principles and approaches to actuate MEMS devices [7, 50–55], the most important of which include: electrostatic actuation [56–64], electrothermal actuation [4, 44, 65–68], electromagnetic actuation [7, 69], and piezoelectric actuation [48, 70] (Fig. 2). Complementary metal–oxide–semiconductor CMOS-MEMS sensor devices are designed to work with either electrostatic actuation using parallel plate capacitors or electrothermal actuation by using microheater. On the other hand, PolyMUMPs and MetalMUMPs sensor devices are designed to use electrothermal actuation by using embedded microheaters [71].

MEMS-based devices could be actuated by applying a sinusoidal force *F*(*t*)*,* through one of the aforementioned actuating methods. Equation (1) is used to find the general equation of motion since *M* is the total mass of the top plate, *b* is the damping coefficient, *k* spring constant of the flexible beam,

1 $$M\ddot{\gamma } + b\dot{\gamma } + k\gamma = F(t)$$

where *F* represents the amplitude of the external driving force in the *z*-direction, $$\ddot{\gamma }$$$$\ddot{\upgamma }$$, $$\dot{\upgamma }$$ $$\dot{\gamma }$$ and *γ* are the acceleration, velocity and displacement of the mass, respectively.

Electrostatic actuators are quite widespread; they have a fast response time and low power consumption [72]. On the other hand, the actuators using electrothermal principle are popular; they have a large driving force and displacement under low driving voltage. Electromagnetic actuators have some merits as a quick response, large displacement. The actuators using piezoelectric principle have high sensitivity although small driving force and displacement per unit voltage. Accordingly, a good understanding of the principle that takes place is essential to obtain a high-performance device with one of these actuation approaches.

### Electrostatic Actuation

**Fig. 3:**
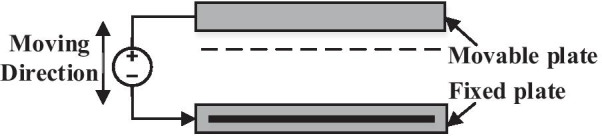
Parallel plate capacitor for the electrostatic actuation [75]

The main source of the electrostatic actuation is the electric field of the capacitor, and it relies on the attractive force between two parallel plates with opposites charges [12]. Electrostatic actuation is based on Coulomb's law which depicts the reciprocal force between two charges with a certain distance that generated between fixed and movable plates. Electrostatic actuation is considered one of the most popular mechanisms for actuating MEMS devices. They have a simple design, fast response time, ease of fabrication and low power consumption. However, the non-linearity and the elevated value of the driving voltage is the major matter of this type of actuator [72]. Some types of electrostatic actuation must be carefully considered when studying MEMS under electrostatic actuation. The most prevalent forms are based on either capacitor consisting of two simple parallel plates or a comb-drive structure consisting of multiple interdigitated or non-interdigitated fingers [72, 73]. A conventional method [74] is the parallel plate actuation in which the top moving plate has a certain polarity, and the bottom fixed plate has an opposite polarity (Fig. 3). The displacement and vibration of the moving plate depend on the voltage difference between the two plates. When the polarity of the charges are similar, there will be a repulsive displacement between the fixed and moving plates, but when the two plates have different charges, the moving plate will be attracted to the fixed plate. Actuation results when the type of charges on the moving plate are changed periodically [50, 56, 74].

**Fig. 4:**
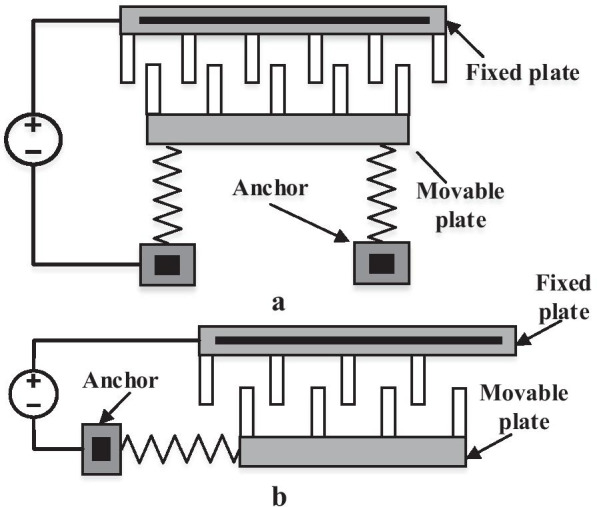
Schematic of the electrostatic comb-drive configurations [84, 85], **a** longitudinal interdigitated comb actuator; **b** transverse actuator

Pull-in instability is one of the main problems when the parallel plate is electrostatically driven because the fixed plate is just below the microbeam and the static friction causes the device to collapse [74, 76]. Comb-drives actuators consist of two comb sets of interdigitated finger structures (Fig. 4). They are commonly placed in the same parallel plate to the substrate where one comb finger is fixed, and the other is connected to flexible structures (e.g., springs) to move toward and away from the fixed plate [72]. When a different voltage is applied between the movable comb and fixed comb fingers, the electrostatic force of the fringe fields attracts the two combs together by a fixed external sinusoidal force. Commonly, parallel plate capacitors are stronger than comb-drive actuators per unit area because they have a greater overlap capacitance between the fixed and moving plate of the actuator. However, designers prefer to comb actuators due to these two main reasons: it produces a larger displacement (a few tens of micrometers are available), and the force is relatively not related to the displacement. Overall, electrostatic actuation has found wide applications in micromechanical actuator [77], biosensor application [56], humidity sensing [57], particle and mass sensing applications [50, 78], MEMS nanopositioning system [58], RF MEMS switch applications [13, 79], closed-loop noise of MEMS oscillators [60], navigation (automobile Global Positioning System GPS) [62, 80], gyroscopes [81], biomarker detection in exhaled breath [82] and mass-sensitive gas sensors [63]. Besides, the theoretical model of electrostatically actuated and capacitive CMOS- MEMS based sensor device for noninvasive screening of diabetes was also reported. Wherein diabetic screening was conducted through detection of acetone vapour in exhaled breath (EB), where chitosan polymer was used as a sensing material. Specifically, an optimized sensitivity was reported about 0.042, 0.066, 0.13, 0.18, and 0.26 pm/ppm [83].

**Fig. 5:**
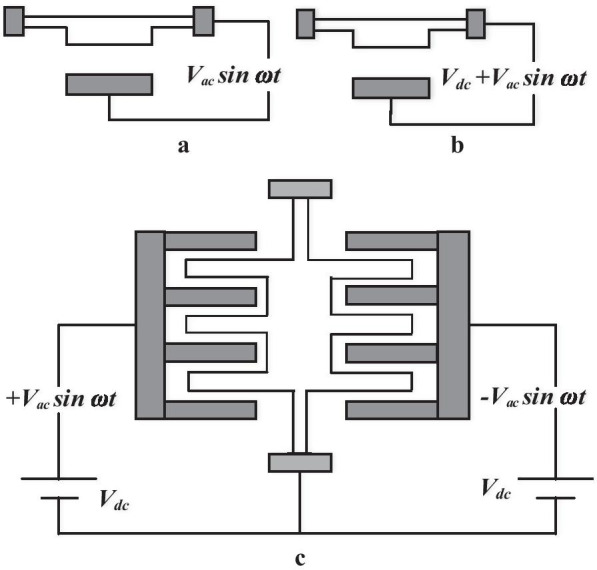
Comb driving resonator **a** Simple alternative voltage driving, **b** alternating voltage with a dc bias, and **c** push–pull driving scheme [86]

In order to excite a MEMS device into resonant condition or vibration mode, an alternative driving must be performed by applying an AC voltage between the two plates of stator and rotor. The standard driving modes are shown as per the following [49]: simple alternative voltage (*V*_1_sin*ωt)*, alternating voltage with a dc bias (*V*_0_ + *V*_1_sin*ωt)* and push–pull driving (Fig. 5). Push–pull driving is generally considered the best solution because structural forces can be applied to both sides of the rotor. Push–pull is an ideal drive appropriate for comb drive scheme [49].

### Electrothermal Actuation

Electrothermal techniques are mostly utilized in MEMS drives while seldom utilized in sensing applications [72]. The electrothermal technique is essentially appropriate for flexible configuration to generate movement with the desired displacement. Driven is conducted by an AC-current applied through the terminals of the embedded microheater owing to the thermal force resulted as a response to the temperature difference of the different layers [66]. The microheater is made of components of different layers of material and operates at elevated temperatures. When the AC current is applied, the temperature of the device rises; owing to the mismatch in the coefficient of thermal expansion (CTE) of the different materials, the material expands, causing thermal stress that causes the device to bend [72]. On the other hand, the cooling approach can be accessible through conduction to the substrate and convection to the surrounding air (or liquid).

**Fig. 6:**
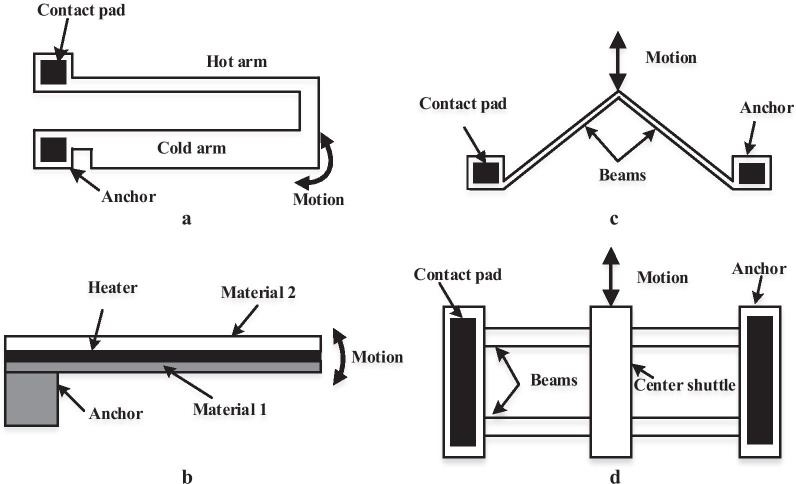
Schematic of the electrothermal actuation **a** U-Beam Actuator (hot/cold arm), **b** bimorph actuator (Bi-material actuator), **c** and **d** buckling actuators (V-Beam Actuator and shuttle beams) [67, 92]

These changes in temperature affect the device in two ways, as reported in [44, 87]: (1) dimensional changes in the device or stresses generated inside the device, and (2) material properties of the device vary with temperature. The current technique needs to be compacted to the microheater. Microheater generates the desired heat owing to applied a specific current to the resistor can result in low power dissipation and quick response time. The electrothermal force can be described by (2) [88].

2 $$F_{th} = \alpha_{T} AE(T - T_{0} )$$

where* α*_T_ is the CTE,* E* is the young's modulus of the beam,* A* is the cross-section area of the beam,* T* is the final temperature, and* T*_0_ is initial temperature before heating. Several materials have been used in microheater designs. For example, to achieve the best performance, accuracy, widespread temperature scope and stability, platinum and gold will be the best decisions for the microheater [89]. However, they are costly [90]. Utilizing microheaters in MEMS sensors need a built-in temperature sensor to convert the produced heat to useful output. For temperature sensors, platinum is available in the − 200 ºC to 800 ºC temperature range and nickel is available in the − 100 to 260 ºC [90]. According to reports, aluminium is also a good material for temperature sensing [91]. Figure 6 shows the common mechanisms of electrothermal actuation that include U-Beam Actuator (hot/cold arm), bimorph actuator (Bi-material actuator), and buckling actuators (V-Beam Actuator or shuttle beams).

U-beam actuators are also known as hot/cold arms, where the actuator is made of a narrow arm (hot), a wide arm (cold) anchor and contact pads [67, 92]. It is primarily utilized for in-plane or horizontal incitation comparative with the substrate. The working principle in this technique depends on applying a differential voltage over the contact pads, current flows through the arms, and the hot arms (higher resistance) heats up more current density than the cold arm (lower resistance). A narrower hot arm is, therefore, hotter and will expand more than a thicker arm. The hot arm produces large thermal stresses. This differential strain between both arms makes the whole device to deflect.

A bimorph actuator, also referred to as a bi-material, consists of two different thermally expanding materials bonded together. Unlike the first two actuators, the bimorph actuator [93] is typically used for out-of-plane actuation. As the current passes through the beam, the temperature of the material increases. This temperature will produce a greater expansion in one material than the other because it has a different thermal expansion coefficient, resulting in thermal stress and bending of the device.

Thermal buckling actuators consist of a V-beam or shuttle beam [94], anchors and contact pads, which are also primarily used for in-plane or lateral actuation. As current flows among the beam, beam temperature rise causes thermal expansion due to the Joule heating that occurs, which tends to expand and move the shuttle or push the centre beam of the device forward in the simplest and less resistant direction.

In the real implementation, the majority of the MEMS devices with thermally actuating are vibrated at frequencies near to the natural frequency, so it is essential to calculate the natural frequency of the devices. The electrothermal technology becomes more prevalent owing to its large driving force and displacements based on thermal expansion of the tow layer materials under a very low excitation voltage, which cannot be accomplished by whatever other strategies when similar measurements are utilized [85, 95]. Electrothermal actuation exhibits multiple advantages such as large displacement [95], ease of fabrication [72], large force, and relatively low applied voltage [52]. On the contrary, they require a large amount of current and a low voltage amplifier. In addition, it consumes high power due to Joule heating. One more limitation is a sensitivity to the ambient temperature. Finally, the exciting temperature and heat increased owing to electrothermal actuation can induce several drawbacks for the neighbouring electronics along with the packaging phase of the system [72].

Numerous types of electrothermal actuation have been developed and studied; Hot/cold arm [96–98], bi-material or bimorph [98, 99] and thermal buckling actuator [98, 100]. Reference [101], has investigated the micro-cantilever vibrations under thermal actuation using the bimorph actuator. Reference [102], has suggested a structured procedure for polysilicon micro ring thermal actuation (RTA) fabricated utilizing the MUMPs processes. A micro-cantilever based on the thermal actuation has designed. They assumed the system is in vacuum or fluid (gas) liquid and suggested an analytical solution [103]. Dennis et al. [44] fabricated a (CMOS-MEMS) system with built-in microheaters operated at relatively high temperatures (40–80 °C) to measure humidity levels using titanium dioxide (TiO_2_) nanoparticles as a sensing material. The sensor was operated in dynamic mode using an electrothermal actuation and an output signal assessed using a piezoresistive (PZR) sensor connected to the Wheatstone bridge circuit. The humidity sensor output voltage rises from 0.585 to 30.580 mV as the humidity increases from 35% RH to 95% RH. The sensitivity of the humidity sensor increases linearly from 0.102 mV/% RH to 0.501 mV/% RH with an increase in the temperature from 40 to 80 °C and a maximum hysteresis of 0.87% RH is found at a relative humidity of 80%. In a further notable contribution, Almur et al. [104] modelled a MetalMUMPs acetone vapour sensing system based on electrothermal actuation and capacitive sensing. The output voltage change was found to increase linearly with increasing the acetone vapor concentration from 100 to 500 ppm with a concentration sensitivity of 0.65 mV/ppm. Due to the thick nickel layer of the MetalMUMPs technology used the device has very high mass (4.7 × 10^–8^ kg) and low mass sensitivity (0.118 MHz/pg).

### Electromagnetic Actuation

**Fig. 7:**
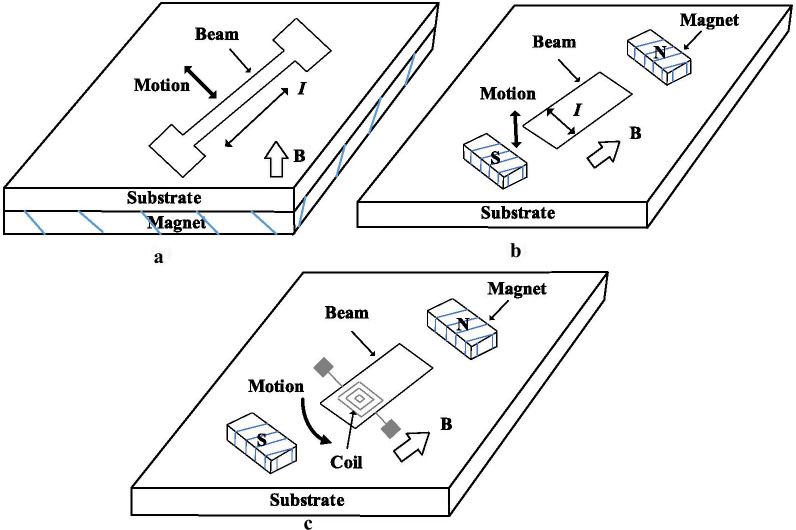
Schematic of the electromagnetic actuation using Lorentz forces **a** in-plane actuator, **b** and **c** out-of-plane actuator [72]

The electromagnetic operation has long been utilized in the sensing and driving of the large scaling applications (such as loudspeakers). This technique demands electricity to stimulate the magnetic effect. It uses the electromagnetic force (also can be called Lorentz force after the Dutch physicist Hendrik A. Lorentz 1895) to produce a magnetic field to the resonator by applying an alternating current on the coil or inductor integrated into the resonator (Fig. 7). Lorentz Force is defined as the force generated on a point charge as a result of the combined electric and magnetic forces on the charge [72]. Electromagnetic actuation has also been utilized as a guide to other actuation techniques, for example, electrostatic [33] and electrothermal [34]. In this technique, when a conductor conveying an electric current *I* can be applied to an external magnetic field *B*, this will induce a Lorentz force *F*_*L*_ as stated by (3) [72].

3 $$\vec{F}_{L} = L\vec{I} \times \vec{B}$$

where *L* indicted to the conductor length and × refers to the cross product operation. The current in the conductive element located within the magnetic field generates an electromagnetic force in a direction perpendicular to the current and the magnetic field. This force has a directly proportional to the current, magnetic flux density and length of the microbeam. Because of the interaction generated by the current between the magnet and the magnetic field, a mechanical displacement on the microbeam will be generated as a result of the applied current to the micro-coil. For example, electromagnetic actuation has been used to stimulate microbeam when being exposed to an external magnetic field by flowing current through the microbeam, as seen in Fig. 7a. Similarly, as alternating current flows through the device on the microbeam, this approach can be used to excite the out-of-plane resonator (Fig. 7b). Lorentz forces of equal value and opposite directions are produced if the coil is deposited on a structure of the microbeam. The opposite directions of the current flowing through the different parts of the coil correspond to these forces. Such forces balance one another out. Figure 7c, however, can induce a net motion that is used to actuate the microbeam [72].

The electromagnetic technique involves many merits over the other kind of actuation techniques. The main advantages of the electromagnetic actuation include the large displacement without the influence of the nonlinear effect [105], lower voltages due to they based on current rather than voltage-driven [7], in addition, they have a high reproducibility rate, they also have more features like rapid response, high accuracy, and the merit for controlling easily. For MEMS applications, the electromagnetic technique is the best choice to achieve maximum driving force for device size ratio [106]. However, they still suffering from some drawbacks due to using the huge current resulting in high power consumption, also their fabrication is complicated (normally be in need of inductive parts to produce magnetic flux also the possibility of including the manufacture of coils, and the deposition of a magnetic material) [7]. This fact combined with constructive difficulties has limited magnetic actuation applications. However, there are successful application examples in the literature as it may exist in MEMS switches devices [7], Optical switches, and micro scanners [69], gyroscopes [107], or relays [108]. Many researches are also based on magnetic actuation [109]. The magnetic microsensors utilizing the commercial 0.35 μm CMOS process has been investigated [110]. In a further contribution, Barba et al. [111] designed an electromagnetically actuated MEMS cantilevers proposed in order to minimize parasitic phenomena using Boron-doped silicon. In another seminal work, an electromagnetic and Piezoelectric actuated and piezoresistive sensed CMOS- MEMS device has been modelled for humidity sensing using ZnO NRs (6 μm/chitosan SAMs) as a sensing material [83]. The sensitivity was found to be 83.3 ppm [112].

### Piezoelectric Actuation

Piezoelectric effect indicates the capability of a particular material to produce an electrical voltage in response to applied the mechanical stress. Piezoelectricity was discovered by the Curie brothers in 1880, and 'Piezo' meaning is coming from the Greek word that is mean 'press' or 'push'. A piezoelectric actuator defined as transducers that converts an applied potential into a mechanical movement or strain based on the piezoelectric influence [72]. Piezoelectric materials like quartz, zinc oxide, lead zirconate titanate (piezoelectric ceramic material PZT), and polyvinylidene difluoride (PVDF) are commonly used in sensor and actuator applications on a macro scale as well as in MEMS applications in recent years, but the sensing is limited due to their lack of a DC response [49]. Piezoelectric materials act electrically as insulators and required to be placed between two conductive electrodes so as to gather charge or to apply an electric-field (using an interdigitated electrode or sandwich structure) [113].

**Fig. 8:**
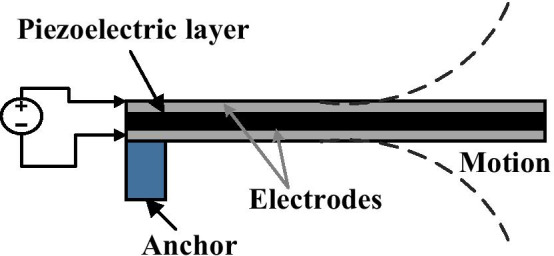
Schematic of the piezoelectric actuator [72]

In MEMS devices, the basic principle of the piezoelectric actuation method is based on using a thin piezoelectric layer that is deposited as a part of the MEMS beam between flexible (Fig. 8). When a voltage is applied to the piezoelectric layer, the piezoelectric material expands according to the polarization of the applied voltage, which causes an axial bending across the length of the flexible beam [114].

In general, piezoelectric actuators exhibit a lot of advantages such as high output force, good operating bandwidth, very compact size, lightweight, low power consumption, and fast response [114, 115]. Their characteristics are well known and have been used for decades. Most first sensors use piezoelectric actuation and are still in use today. However, small displacements of actuators are a major drawback, and their high-temperature sensitivity [72], nonlinear working area and hysteresis limit their wide range of applications [49]. Piezoelectric materials are very brittle. They can seldom be utilized alone but are often connected to a flexible structure for actuation and sensing applications. There are several works based on piezoelectric actuation [2, 48, 114, 116] studied the prospective to use a piezoresistive microcantilever for environment application, particularly for humidity sensing. Mahdavi et al. [117] proposed a new class of accurate dew point measurements taking advantage of thin-film Piezoelectric-on-Silicon (TPoS) resonators using ZnO NRs (6 μm/chitosan SAMs). The sensitivity was found to be 16.9–83.3 ppm at the range of response time of 46 s/167 s. sensitivity to mass loading based on piezoelectric excitation is investigated for chemical sensing [118, 119] reported piezoelectric transduction of flexural-mode silicon resonators to achieve efficient temperature compensation. The resonance gas sensor using piezoelectric MEMS for defence applications was reported [120].

### Actuation Techniques Discussion

Different types of MEMS actuators require different drive electronics. As per mentioned in this article, there are different principles and approaches to actuate MEMS devices. The most important of which include: electrostatic actuation, electrothermal actuation, electromagnetic actuation, and piezoelectric actuation. Electrostatic actuators are a traditional field and considered one of the most popular mechanisms for actuating MEMS devices. They have a simple design, fast response time, ease of fabrication and low power consumption. However, the non-linearity and the elevated value of the driving voltage is the major matter of this type of actuator. Electrothermal actuators provide very low resistance to their drive sources and require high currents rather than high voltages to move them. High voltage amplifiers are not particularly suitable for use in electrothermal MEMS actuators. Instead, a low voltage and high current signal source are required. Of course, this can also work if the output current of the high voltage amplifier is large enough to drive the electrothermal actuator, but it is not a particularly energy-efficient or cost-effective solution. For MEMS applications, electromagnetic technology is the best choice to achieve maximum driving force per device size ratio. However, they still suffering from some drawbacks due to using the huge current that results in high power consumption, also their fabrication is complicated (normally be in need of inductive parts to produce magnetic flux also the possibility of including the manufacture of coils, and the deposition of a magnetic material). Piezoelectric actuators also required high voltage amplifiers, but since they are typically used for resonance, the drive requirements in terms of driving voltage are typically lower than those required for other actuators. The recent research of MEMS-based sensor devices which can be actuated using the four basic actuation techniques that have been used during the last few years for many applications, as shown in Table 2. It can be clearly observed that there is a rising development pattern in the field of micro-assembly and micromanipulation for MEMS-based sensor devices that have been categorized based on actuation techniques. Nowadays, a wide range of applications has been identified for MEMS-based sensor devices that provide high sensitivity and resolution. The sensing systems are getting more robust and reliable due to the integration configuration.

**Table 2:** Summary of actuation mechanisms for MEMS-based sensor devices

Actuation technique	Technology/device	Operation principle	Applications	Year	Refs.
Electrostatic	BioMEMS sensor	Capacitive based Interdigitated electrodes (IDE)	Biosensor application	2019	[56]
Electrostatic	Microcantilever -based MEMS sensors	Capacitive sensing	Humidity Sensor	2019	[57]
Electrostatic	MEMS nanopositioning system	Sigma-delta type arrangement	Nanopositioner	2016	[58]
Electrostatic	Bistable MEMS switch	Pull-in voltage	Switch applications	2016	[59]
Electrostatic	MEMS resonators	Parallel-plate model	closed-loop noise of MEMS oscillators	2016	[60]
Electrostatic	MEMS gyroscope	Comb drives or parallel plate capacitors	Navigation (automobile GPS)	2016	[62]
Electrostatic	CMOS-MEMS resonator	Interdigitated comb actuator	Mass-Sensitive Gas Sensors	2014	[63]
Electrostatic	CMOS-MEMS resonator		Biomarker Detection in Exhaled Breath	2015	[82]
Electrothermal	MEMS-based acetone vapour sensor	Different coefficients of thermal expansion (CTEs)	Diabetes screening	2018	[66]
Electrothermal	CMOS-MEMS device	Different CTEs	Humidity sensing purpose	2017	[4]
Electrothermal	MEMS resonator based filters	U-shape double-clamped beam	Filter components	2016	[67]
Electrothermal	CMOS-MEMS sensor	Different CTEs	Humidity Sensing	2015	[44]
Electrothermal	Tuneable MEMS ring resonator	Different CTEs of materials	Filtering Applications	2015	[68]
Thermo-Electric	MetalMUMPs device	Different CTEs of materials	Out-of-Plane micrometric Displacements	2014	[121]
Thermal actuation	A MEMS-based electrostatic field sensor (EFS)	Clamp-clamp beam	Modulate the external electrostatic field	2014	[122]
Electromagnetic	MEMS actuator	Lorentz force	Optical switches, and micro scanners	2015	[69]
Piezoelectric	Mass-sensor based micro-cantilevers	ZnO thin films	A bio-or chemical event detection	2019	[48]

### Sensing Mechanisms for the Output Signal

The sensing mechanism is used to sense the output of the MEMS devices. The basic of the sensing technique is based on the use of a polymer deposited on the sensing layer of the device to absorb a particular chemical that creates a variation in the stress, mass, electrical or mechanical characteristics of the beam. To estimate the change in the mass, the resonant frequency of the device can be measured based on the detected mass increment that is one of its parameters. Then again, when the polymer absorbs the mass, piezoresistor that is situated on the surface of the sensing element can be used to estimate the mass of the beam. Another method of detecting suspected chemicals is to use thermocouples to measure temperature changes caused by the heat generated by the polymer absorbing analyte [49].

**Fig. 9:**
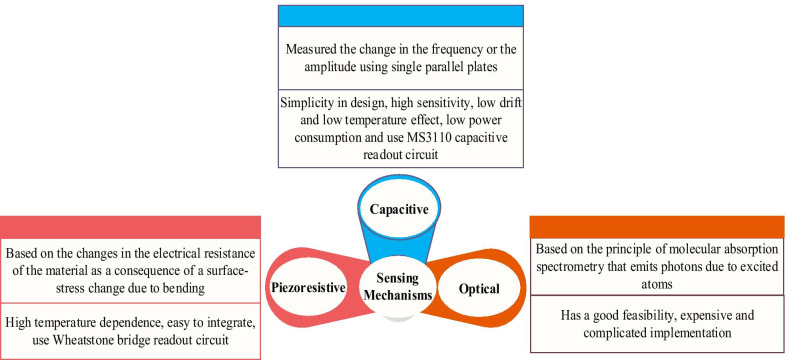
Sensing mechanisms of MEMS-based sensor devices

To sense the output of these MEMS devices methods such as piezoresistive, capacitive or optical mechanisms are used (Fig. 9) [123]. Different sensing mechanisms have different advantages and disadvantages at the same time.

For instance, optical sensing has good feasibility; however, it is very expensive to implement compared to capacitive and piezoresistive techniques [124]. Capacitive and piezoresistive techniques are the common methods used to sense the output signal. CMOS- MEMS device was designed to encompass both piezoresistive and capacitive sensing techniques due to the features of 0.35 µm CMOS technology used to design it. In contrast, PolyMUMPs and MetalMUMPs devices were designed to use only capacitive sensing techniques.

### Piezoresistive Sensing

Piezoresistive is a common sensing principle used in MEMS devices. Essentially, the principle of piezoresistive materials is based on the resistance change when their strain changes in response to applied stress. This change can be observed in the electrical resistance of the material of the device as a consequence of a surface-stress change due to bending. The resistance value of the piezoresistor with the resistivity ρ of a resistor, length of l and cross-section area A is given by (4) [29].

4 $$R = \frac{\rho l}{A}$$

**Fig. 10:**
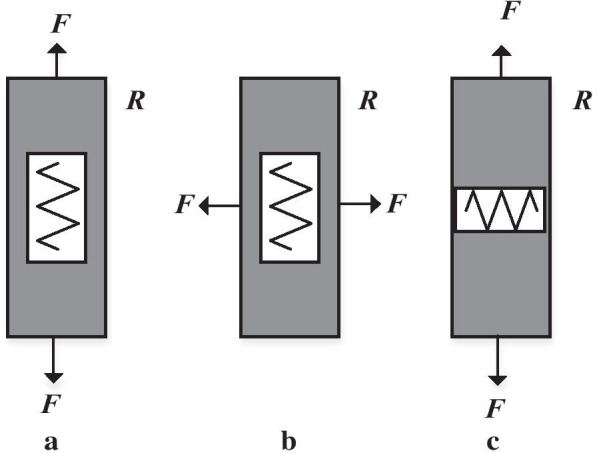
**a** Longitudinal piezoresistance dominates, **b** and **c** transverse piezoresistance dominates presented [29]

The change ΔR in resistance is proportional to the applied strain can be defined in (5) [29, 125].

5 $$\Delta R = G\varepsilon R$$

where G is the gauge factor work as a proportionality constant of the piezoresistor, ε is the strain in the material, and R is the piezoresistor resistance. A piezoresistive element behaves differently towards longitudinal and transverse strain component presented by [29]. According to (5), the resistance of a piezoresistor can vary owing to the changes of the geometry (length and cross-section) or in the property of the material (resistivity). Piezoresistive detection depends on the difference in resistivity of material when stress is applied. In a piezoresistive material, the change of resistivity because of the application of stress has a much greater effect on the resistance than the change in resistivity because of a change in the geometry (sensors rely on the change of resistance owing to the geometry are so-called strain gauges) [72]. (Fig. 10) shows different resistor orientations and external force loading directions [88].

**Fig. 11:**
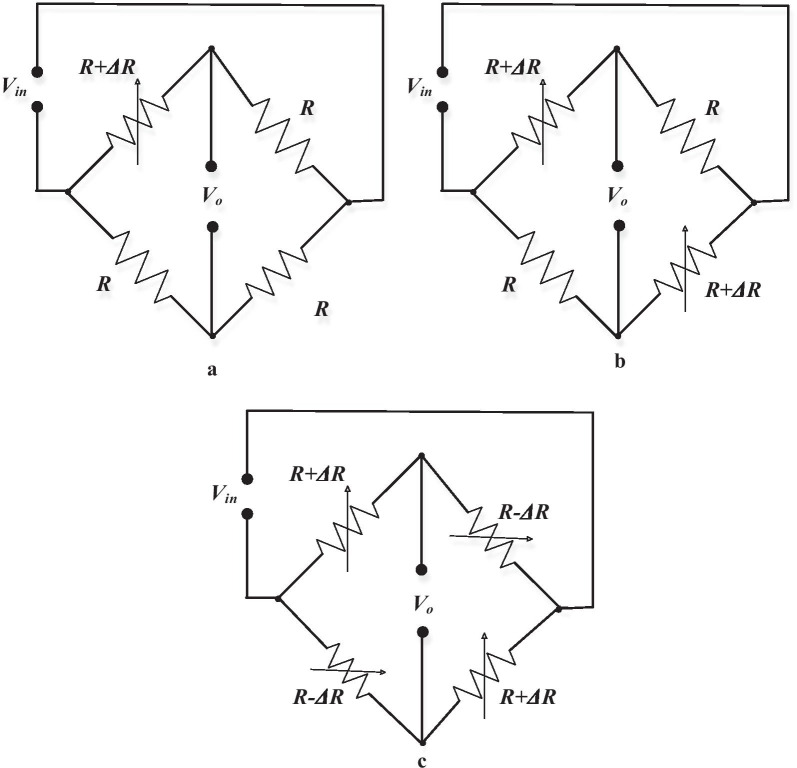
Schematic types of a Wheatstone bridge, **a** quarter bridge, **b** half-bridge, and **c** full-bridge configurations [72]

The main drawback of this sensing mechanism is the solid temperature reliance of resistivity. To limit this impact, normally, a collecting of four piezoresistive designed is utilized to shape a Wheatstone bridge. This limits the temperature reliance [72]. Furthermore, the small changes in the electric signal generated from gauges are translated and gauged as voltages utilizing this Wheatstone bridge [49]. Wheatstone bridge utilizes four resistors arranged in the bridge of the four arms (Fig. 11). Based on the quantity of these active resistors (acting as a transducer), single active bridge (single transducer), half active bridge (double transducers), and the full active bridge (quadruple transducers) of the bridge of Wheatstone might be utilized as reported [72]. The single active bridge as well called Quarter Bridge that utilizes just a single active component which changes because of a physical ambient, for example, pressure, power, temperature, while the remnants of that resistors will be passive and so on. MEMS systems have been used as temperature, calorimeter, and in combination with the Wheatstone bridge, sensors allow the base fluctuations. Thermal bridge system is one of the best-known sensing platforms among MEMS. In the AC-driven Wheatstone bridge configuration (Fig. 11b), researchers have utilized the resistance-based thermometry to analyse temperature changes with micro-Kelvin resolution. In an overview, the right half of the bridge comprises a sensing resistor on the lower branch with associated resistance on the top, while the left side is defined as the matching one. Fixed resistors with a relatively low resistance coefficient of temperature are used with resistance values, which was chosen to enhance stability and resolution based on the previous study [126].

Silicon piezoresistor and polysilicon patches are generally utilized in MEMS sensors. The resistivity of silicon relies upon its strategy for doping. N-type doped with silicon is less sensitive than p-type. This raises limitation to the fabrications. In this situation, an in-plane transversal and in-plane longitudinal will be the most significant elements [72]. For semiconductor materials like silicon, the longitudinal and transverse gauge factors owing to geometry change are quite tiny compared to their values due to resistivity change, hereafter the change in geometry can be ignored, and the longitudinal and transverse gauge factors due to only the resistivity change are found using (6) and (7), respectively [127].

6 $$G_{L} = \pi_{L} E$$

7 $$G_{T} = \pi_{T} E$$

where *E* is Young's modulus of the piezoresistive material. *π*_L_ and* π*_T_ are the longitudinal and transverse piezoresistance coefficients, respectively, for arbitrarily oriented polysilicon grains, and their values are given in Table 3 [19].

**Table 3:** Longitudinal and transverse piezoresistance coefficient of polysilicon [19]

Parameter	P-type polysilicon	n-type polysilicon
*π*_L_	58.8 × 10^–11^/Pa	− 45.4 × 10^–11^/Pa
*π*_T_	− 18.5 × 10^–11^/Pa	34.5 × 10^–11^/Pa

Currently, the piezoresistive effect has been utilized as a bio-or chemical detection [48], humidity sensing purpose [4], switch applications [59], biomarker detection in exhaled breath [82], micro scanners [69], pressure sensing [128], and mass-sensitive gas sensors [63]. Zope et al. [117] developed a resistively sensed thermally-driven piezo resonator composed predominantly of CMOS material for mass sensing applications. whereas mass sensitivity of 24.96 kHz/ng was reported. The extracted mass resolution of 16.3 fg have also been observed, hence showing great potential to serve as an aerosol sensor).

### Capacitive Sensing

The capacitive sensing mechanism is based on measuring the changes of the capacitance between the stator and rotor fingers or between the fixed plate and movable plate [88]. Capacitive sensing is the main dominant method for micromachined applications due to its compatibility with all the fabrication approaches and stiffness [129, 130]. Capacitive sensing has many attractive features include high sensitivity, low power consumption, simplicity in design, low drift and low-temperature dependency. Furthermore, the measurement of the output signal can be easily fabricated on the PolyMUMPs die using capacitive sensing. Only a single parallel plate can be used to capacitively sensed the variations in the natural frequency or amplitude of the MEMS devices (such as a CMOS-MEMS device and a PolyMUMPs device) or by applying a comb finger (such as a MetalMUMPs device).

**Fig. 12:**
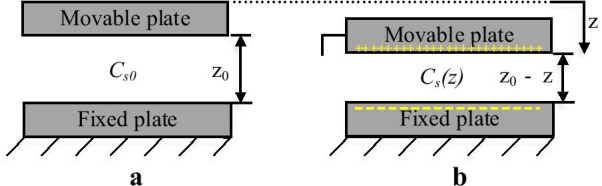
Parallel plate capacitor showing **a** initial condition and **b** after displacing the movable plate [19]

For CMOS-MEMS device and PolyMUMPs devices, when there is no actuation (Fig. 12a), the initial static sensed capacitance (C_s0_) between the lower fixed plate and the movable top plate is calculated using (8) [19].

8 $$C_{s0} = \frac{{\varepsilon A_{s} }}{{z_{0} }}$$

where ℇ is the relative permittivity of the dielectrics, A_s_ is the area of the sensed plates and z_0_ is the gap between the fixed and movable plates. When the movable plate is displaced by z (Fig. 12b) the initial gap changes, and hence the capacitance will change too, and it is found by (9).

9 $$C_{s} (z) = \frac{{\varepsilon A_{s} }}{{z_{0} - z}} = C_{s0} \frac{{z_{0} }}{{z_{0} - z}}$$

**Fig. 13:**
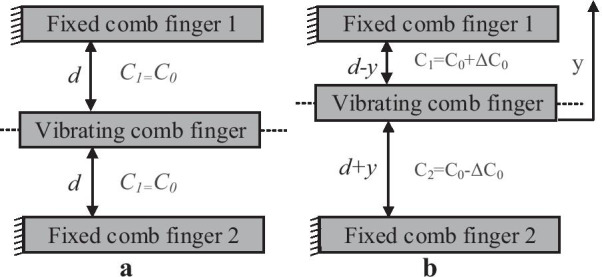
Comb fingers configuration for MetalMUMPs device showing **a** the initial condition and **b** the displacement of the vibrating comb finger [19]

At the same time, for the MetalMUMPs devices, the output can be detected capacitively by utilizing the differential comb fingers design. As appeared in Fig. 13, there are two arrangements of fixed comb fingers to have the differential capacitance.

For the initial condition, when the device is not actuated (Fig. 13a), the static detected capacitance C_0_ for every set of vibrating-fixed fingers could be calculated by (10).

10 $$C_{0} = n\frac{{\varepsilon l_{f} w_{f} }}{d}$$

where* l*_f_ and* w*_f_ are length and width of the overlapping area of the vibrating and fixed comb fingers, respectively. *d* is the gap between the fixed and vibrating comb fingers and *n* is the number of the vibrating comb fingers. When the device is moved by *y* toward the fixed comb finger one as appeared by (Fig. 13b), the capacitances *C*_1_ and *C*_2_ will be changed and relying upon that shift, and their values can be calculated using (11) and (12), respectively.

11 $$C_{1} (x) = n\frac{{\varepsilon l_{f} w}}{d - y}$$

12 $$C_{2} (x) = n\frac{{\varepsilon l_{f} w}}{d + y}$$

The output signal of the device will be measured as a voltage due to change in the sensing capacitance upon actuation using MS3110 capacitive readout circuit that translates the change in capacitance to the output voltage change in response to the temperature of the microheaters. Nowadays, capacitive effects are used in MEMS resonators [60], biosensor application [56], humidity sensor [57], diabetes screening [66], Navigation (automobile Global Positioning System (GPS)) [62], a low noise accelerometer [131], and RF MEMS capacitive switches [132].

### Optical Detection

**Fig. 14:**
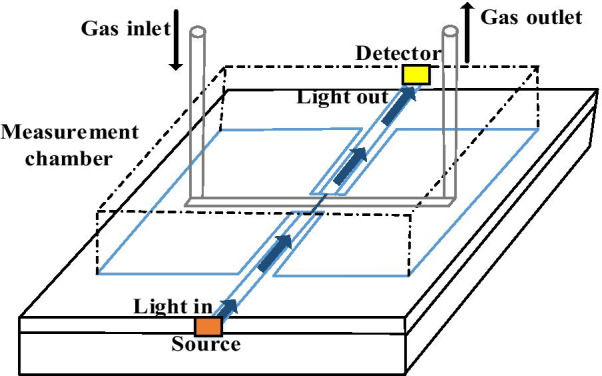
Schematic representation of an optical MEMS sensor [133]

Optical sensing mechanism depends on detecting the changes in the transmitted light compared to the received light. The detection of compound species by spectral transmission strategy is broadly acknowledged. Optical sensors are notable due to their selectivity since it does not rely upon any chemical reaction or any chemical catalyst activities. Air pollutants identification is recognized by using the characteristics of the optical species (for example, absorption, refractive index, Raman scattering, fluorescence, and reflection). Optical sensors utilize emission and absorption measurements primarily through different technologies such as Fourier transform infrared spectroscopy (FTIR), surface plasma resonance (SPR), differential optical absorption spectroscopy (DOAS), laser diode absorption spectroscopy (LDAS), cavity ring-down spectroscopy (CRDS), non-dispersive infrared (NDIR) spectroscopy, light detection and ranging (LIDAR), UV fluorescence and chemiluminescence for the sensing of air pollutants in air specimens. Normally, the light will be cross through, modified or reflected by some space having the relevant medium (Fig. 14). Beer–Lambert law can be used to govern the optical detection of the air pollutants as per the following:

13 $$I = I_{0} *e^{ - \alpha l}$$

where the transmitted light through the specimen is *I*, *I*_*0*_ is the received light, α represent the absorptivity, *l* represents the length of the path.

However, the majority of the applications for optical MEMS are in communications; there are various uses in different fields [134]. The literature covered numerous attempts to introduce the optical detection of air pollutants. A few of these sensors are used to recognize chemicals. This displays the multifunctional part of these sensors. Infrared spectrometry is a case of a technique that can be utilized for optical sensing [135]. In a further notable contribution, Huang and co-workers have also demonstrated [136] the simulation of an integrated optical MEMS accelerometer. The effective simulation results have demonstrated as follows: a resonance frequency of 562.85 Hz, a mechanical sensitivity of 781.64 nm/g, an optical system sensitivity of 1.23, a resolution of 1.037 µg, and low cross-sensitivity. The proposed accelerometer can be used for improvements in MEMS inertial navigation devices. Another model is the Fabry–Perot optical sensor [137]. Basically stated, the principles of this sensor are based on bringing gas into a chamber and a short time later going light through the chamber. Due to changes in the substance piece, the light experiencing will be not exactly equivalent to without the gas. This light is broke down, and from the results, confirmation can be made about the sort of gas being tested. The fruitful testing of an optical MEMS sensor for the location of catechol was reported [133]. The absorbance measurement of catechol oxidation was performed by blue light (472 nm) attached through a MEMS device. The light was transmitted from a free-space blue laser working in persistent wave mode and is focused on a multimode fibre utilizing manually arranged to organize. Received light was coupled by means of the optical fibre to a USB connected spectrophotometer which assisted computerized information collection utilizing the software.

### Sensing Mechanisms Discussion

In view of different working principles, the above-mentioned sensing mechanisms can be divided into three types, namely piezoresistive, capacitive and optical mechanisms. There are benefits and drawbacks for different sensing mechanisms at the same time. For instance, optical sensing has good feasibility; however, it is very expensive to implement compared to capacitive and piezoresistive techniques. As a result, piezoresistive and capacitive techniques are the common methods used to sense the output signal. Any small change in the stress could be detected using a piezoresistive technique. A main disadvantage of the piezoresistive sensing technique is the high-temperature reliance of resistivity. In Table 3, present the recent work and progress (last 5 years), including actuation and sensing mechanisms, sensing materials, fabrication technology and application. The miniaturized microchip presented (in Table 4) can be utilized for gas and humidity sensing, human health screening, inertial navigation with good repeatability, high resolution, high sensitivity, and rapid response. In addition, MEMS sensors have been proven to be an effective medium for sensors combined with on-chip electronic circuitry. It can therefore function not only as a portable sensor chip but can also be linked as part of the Internet of Things (IoT) network to achieve real-time and remote high-sensitivity moisture tracking.

**Table 4:** State-of-the-art actuation and sensing mechanisms for MEMS-based sensor devices (N.M not mentioned)

MEMS device	Dennis et al., 2015 [44]	Dennis et al., 2016 [83]	Almur et al., 2017 [104]	Barba et al., 2018 [111]	Mahdavi et al., 2018 [117]	Jiushuai et al., 2019 [112]	Huang et al., 2019 [136]	Zope et al., 2020 [138]
Actuation mechanism	Electrothermal	Electrostatic	Electrothermal	Electromagnetic	Piezoelectric	Electromam gnetic and piezoelectric	N.M	Thermal
Sensing mechanism	Piezoresistive (PZR)	Capacitive	Capacitive	N.M	Thin-film piezoelectric-on-silicon	Piezoresistive	Optical	Piezoresistive
Resonance frequency	2–20 Hz	100–100 kHz	0.5–8 kHz	6.5 kHz	18–20 kHz	18–20 kHz	562.85 Hz	5.13 MHz
Sensitive material	Titanium dioxide (TiO_2_) nanoparticles	Chitosan	Chitosan/polyethylene glycol (PEG) polymers	Boron-doped silicon	ZnO NRs (6 μm/chitosan SAMs)	ZnO NRs (6 μm/chitosan SAMs)	Silicon	Silicon dioxide (SiO_2_)
Sensitivity	0.102 mV/% RH to 0.501 mV/% RH	0.042, 0.066, 0.13, 0.18, 0.26 pm/ppm	0.65 mV/ppm	N.M	16.9–83.3 ppm (30–80%RH, 25 C)	16.9–83.3 ppm (30–80%RH, 25 C)	781.64 nm/g	24.96 kHz/ng
Response time	N. M	N.M	N.M	N.M	46 s/ 167 s	46 s/ 167 s	1.037 μg	7.3 μs
Fabrication technology	CMOS-MEMS	CMOS-MEMS	MetalMUMPs	SOI	N.M	CMOS-MEMS	Bulk silicon process	CMOS-MEMS
Application	Humidity sensing	Screening of diabetes	Acetone vapour sensing	Minimize parasitic phenomena	Dew point meters	Humidity sensing	Inertial navigation	Mass sensing

## Discussion

Recent developments are about new technologies that can harvest energy from the environment, because sustainable self-sufficient micro/nano power sources are an emerging field of nanoenergy, which involves nanomaterials and nanotechnology when harvesting energy for powering micro/nano systems [139]. The triboelectric charge is produced only on the surface dependent on the physical friction between two separate materials. The triboelectric nanogenerator (TENG) is a device that transforms mechanical energy into contact separation or relative sliding between two materials with opposite polarities. TENG based on the coupling effect between triboelectricity and electrostatic induction in which it’s updated progress and potential applications as new energy technology and as self-powered active sensors. The suggested approach uses biomechanical energy transfer to electricity from human activity [139, 140]. Amongst them is a successful study has been investigated for the conversion to wearable energy from portable biometric devices and self-powered sensors based on triboelectricity (i.e. the charge produced on the touch surface) [141–144].

- To decrease power consumption during operation, sensors of woken up or almost zero-power supplement need to proposed and investigated, meaning that these sensors do not have any energy consumption. With these components of MEMS, MEMS sensors can work for a long period or can be operated in a self-powered.
- Looking forward to the future-oriented sensors, sensors with flexibility have become hot topics of the recent research in the latest years owing to their versatility and great prospective in health/human beings applications. Flexible sensors are often used in combination with wearable sensors to have their unique advantages. More than just utilizing the flexible sensor such as a wearable electronic for observation functions, they can be even utilized as a human–machine interface for achieving higher requirements. With the enhancement of the quality of human life, wearable devices and human–machine interfaces have been recognized as important directions for developing sensors of the future with sufficient flexibility and versatile sensing capabilities. Wearable electronic devices can be simply integrated with the human body to extend our perception capabilities. Sensor functions of wearable electronic devices include, but are not limited to, force, strain, electrophysiology, heart rate monitors, temperature, fitness trackers, etc. With the service of various devices among different anatomical positions, development of many applications of the human body sensor can be facilitated from hospital care to fitness and wellness tracking, human–machine interfaces and recognition and assessment of cognitive states.
- Meanwhile, by combining MEMS sensor with artificial intelligence (AI), the next generation of sensors will provide clear evolutionary impact and help humans interact with other things in various applications circumstances. Moreover, the quick improvement of the modern community has observed the expanding association among people with machines, demanding huge intelligent human–machine devices. Around a huge data and hypersensitive detecting, MEMS sensor utilizing a machine learning strategy dramatically stimulates the enlargement of the coming generation smart sensing system. This interactive system with next-generation sensor provides consumers with a more comprehensive experience. It can be used for many practical applications, such as simulation of sports training, entertainment, medical rehabilitation and so on.

## Conclusions and Future Trends

It is well recognized that MEMS-based sensors play a key role in the field of miniaturization and electronic microcircuits. It is necessary to understand and compare these mechanisms since they are the basis of the MEMS-based devices operation and output signal detection**.** This article reviews several popular actuation and sensing mechanisms related to MEMS devices that have emerged over the past few decades. This article introduced a descriptive overview to the advancement of the actuation and sensing mechanisms of the MEMS-based sensor devices. The up-to-date trends and the restrictions while giving a valuable perception into the field of emerging actuation and sensing technologies. A comprehensive discussion was presented, underlining the significance of the actuation and sensing mechanisms, its structure, working principles, classification, fabrication and applications. Proper understanding of the actuating and sensing mechanisms for the MEMS-based sensor devices play a vital role in their selection and effective application in various innovative technologies. In addition, the proper selection of actuating and sensing techniques in MEMS sensors based on the desired application such as sensitivity, resonant frequency, input\output voltage, temperature etc., will lead to fast-commercialization and better product stability.

In order to perform various tasks for different applications such as industry and electronic equipment, different MEMS devices with different structures collaborated with diverse actuation and sensing mechanisms will arise in true-life. Moreover, MEMS devices will not only be used for simple tasks, but also for more jobs that are complex. At the same time, MEMS devices will have greater freedom and can meet the needs of a variety of applications. However, in order to pursue higher accuracy and miniaturization, the complexity of MEMS device design will continue to increase. In addition, MEMS products have a close relationship with the market. It can be predicted that MEMS devices will realize more functions, miniaturization and low cost, which is a huge challenge for other products. Therefore, in the next few years, MEMS fabrication approaches will develop to a higher level to meet market demand. With the advancement of MEMS device fabrication processes, unique hardware makers will seek after shorter fabrication cycles and quicker fabrication speeds.

One of the central areas of the future trend in this area is reliability research and improvement methods. Moreover, new materials and cost-effective of the fabrication techniques will provide important opportunities for development. It has been demonstrated that the design capability is not limited to standard principles and can improve qualitative performance. Despite MEMS advantages, few challenges still exist in the following aspects.

- The traditional MOS thin films by MEMS techniques often show poor sensitivity to target gases due to the compact surface structure and low crystallinity.
- In recent studies, it have tried to integrate high-performance MOS nanomaterials onto microheaters. Still, it is difficult to control and cast the slurry-based MOS nanomaterials onto the suspending heating area of microheaters. However, the low yield and large device-to-device deviation hamper the sensor fabrication on a large scale.
- It is also complicated to improve the adhesion between microheaters and sensing materials to get stable parameters, especially at high temperatures > 350 °C which results in decreased sensing performance and low stability [14, 145].
- Fabricating sensing films with high sensitivity using MEMS compatible methods is an urgent goal.
- Towards a new era, different challenges can face MEMS-based sensors in terms of power supply and intelligence during the operation, such as the challenges of the flexibility of wearable applications, the friendly interactive capabilities of human–machine interface applications, and analysis of the huge data.

## Data Availability

Not applicable.

## Abbreviations

MEMS: Microelectromechanical system
pH: Potential hydrogen
ICs: Integrated circuits
LOD: Limit of detection
MUMPs: Multi-user MEMS
MST: Microsystems technology
RF: Radio frequency
CMOS: Complementary metal oxide semiconductor
CTE: The coefficient of thermal expansion
AC: Alternating current
DC: Direct current
RTA: Ring thermal actuation
PVDF: Polyvinylidene difluoride
GPS: Global positioning system
FTIR: Fourier transform infrared spectroscopy
SPR: Surface plasma resonance
DOAS: Differential optical absorption spectroscopy
LDAS: Laser diode absorption spectroscopy
CRDS: Cavity ring down spectroscopy
NDIR: Non-dispersive infrared
LIDAR: Light detection and ranging
IDE: Interdigitated electrodes
PZR: Piezoresistive
EFS: Electrostatic field sensor
AI: Artificial intelligence
